# High-mobility group box 1 facilitates migration of neural stem cells via receptor for advanced glycation end products signaling pathway

**DOI:** 10.1038/s41598-018-22672-4

**Published:** 2018-03-14

**Authors:** Xin Xue, Xingxing Chen, Weili Fan, Guan Wang, Liang Zhang, Zongfeng Chen, Peng Liu, Mingyong Liu, Jianhua Zhao

**Affiliations:** Department of Spinal Surgery, Daping Hospital, Research Institute of Surgery, The Third Military Medical University, Chongqing, China

## Abstract

High-mobility group box 1 (HMGB1) facilitates neural stem cells (NSCs) proliferation and differentiation into neuronal linage. However, the effect of HMGB1 on NSCs migration is still elusive. The present study is to investigate the corelation between HMGB1 and NSCs migration and the potential mechanism. The results indicated that 1 ng/ml HMGB1 promoted NSCs proliferation using CCK8 assays. Moreover, data showed that 1 ng/ml HMGB1 facilitated NSCs migration via filopodia formation using phase-contrast and transwell assays. Furthermore, 1 ng/ml HMGB1 upregulated the expression of RAGE, one of the HMGB1 receptor, using western blotting assays and immunofluorescence staining. In addition, 1 ng/ml HMGB1 increased the percentage of filopodia formation using phalloidin staining. Meanwhile, the enhanced migration effect could be abrogated by 50 nM FPS-ZM1, one of the RAGE antagonist, and RAGE-specific siRNA through immunofluorescence and phalloidin staining. Together, our data demonstrate that HMGB1/RAGE axis facilitates NSCs migration via promoting filopodia formation, which might serve as a candidate for central nervous system (CNS) injury treatment and/or a preconditioning method for NSCs implantation.

## Introduction

Neural stem cells (NSCs), one subtype of stem cells, hold promising regenerative therapeutic strategy as their capacity for self-renewal, migration toward lesions and differeniation into neurons, astrocytes and oligodendrocytes to restore the injuried regions following brain and spinal cord injury^1–3^. Given that the activation of endogenous NSCs is limited, transplantation of exogenous NSCs is an effective alternative strategy. Especially, recent studies have indicated the implantation of NSCs into lesions after central nervous system (CNS) injury helps rebuild damaged tissue and facilitate functional rehabilitation *in vivo*^4–6^. Researches have showed that NSCs engraftment promotes neuroplasticity, local inflammation repression and neurovascular reconstruction after brain attack^7^. However, studies have also proved that transplanted NSCs are not sufficient or incapable to survive for a long time in lesions^8–10^. Hence, it is essential to develop strategies to maitain quantitative and qualitative NSCs after transplantation.

High-mobility group box 1 (HMGB1), one of inflammatory proteins exerting beneficial effects at chronic phase following injury, is a highly conserved, nonhistone nuclear DNA-binding protein widely expressed in most mammals, and it contributes to the architecture of chromatin^11^. Specially, previous studies have revealed endogenous HMGB1 promotes vascular remodeling to support the functional recovery following intracerebral hemorrhage (ICH)^11,12^. Furthmore, extracellular HMGB1, which serves as a macrophage-activating factor, could bind to receptor for advanced glycation end products (RAGE) and activate the proliferation of endothelial cells^13^, as well as induce endothelial cell migration and sprouting in tumor progression and propagation^14^. Meanwhile, HMGB1, which produced by local reactive astrocytes after injury, promotes neurovascular repairment after cerebral ischemia^15,16^. In addition, HMGB1 promotes adult hippocampal neural progenitors differentiation into neurons in Alzheimer’s disease^17^ and facilitates functional recovery, but not exacerbating inflammation via RAGE/Nuclear Factor-kappa B (NF-κB) axis after spinal cord injury^18^. Considering that HMGB1 holds the ability of inducing NSCs proliferation and differentiation, migration is another important factor for NSCs to restore lesions after injury. Hence, it is necessary to investigate the corelation between HMGB1 and NSCs migration to further uncover the role of HMGB1 in NSCs.

In the present study, we examined the hypothesis that HMGB1 promoted NSCs migration, and investigated the possible intracellular signaling pathways in this process. Subsequently, FPS-ZM1 and RAGE RNA interference (RNAi) were used to evaluate the effects of HMGB1/RAGE axis on NSCs migration *in vitro*. The present study finds an experimental answer to explain the potential mechanism through which NSCs spontaneously migration towards the lesions and might provide a suitable candidate following brain and spinal cord injury.

## Results

### HMGB1 significantly enhanced proliferation of NSCs

**Figure 1:**
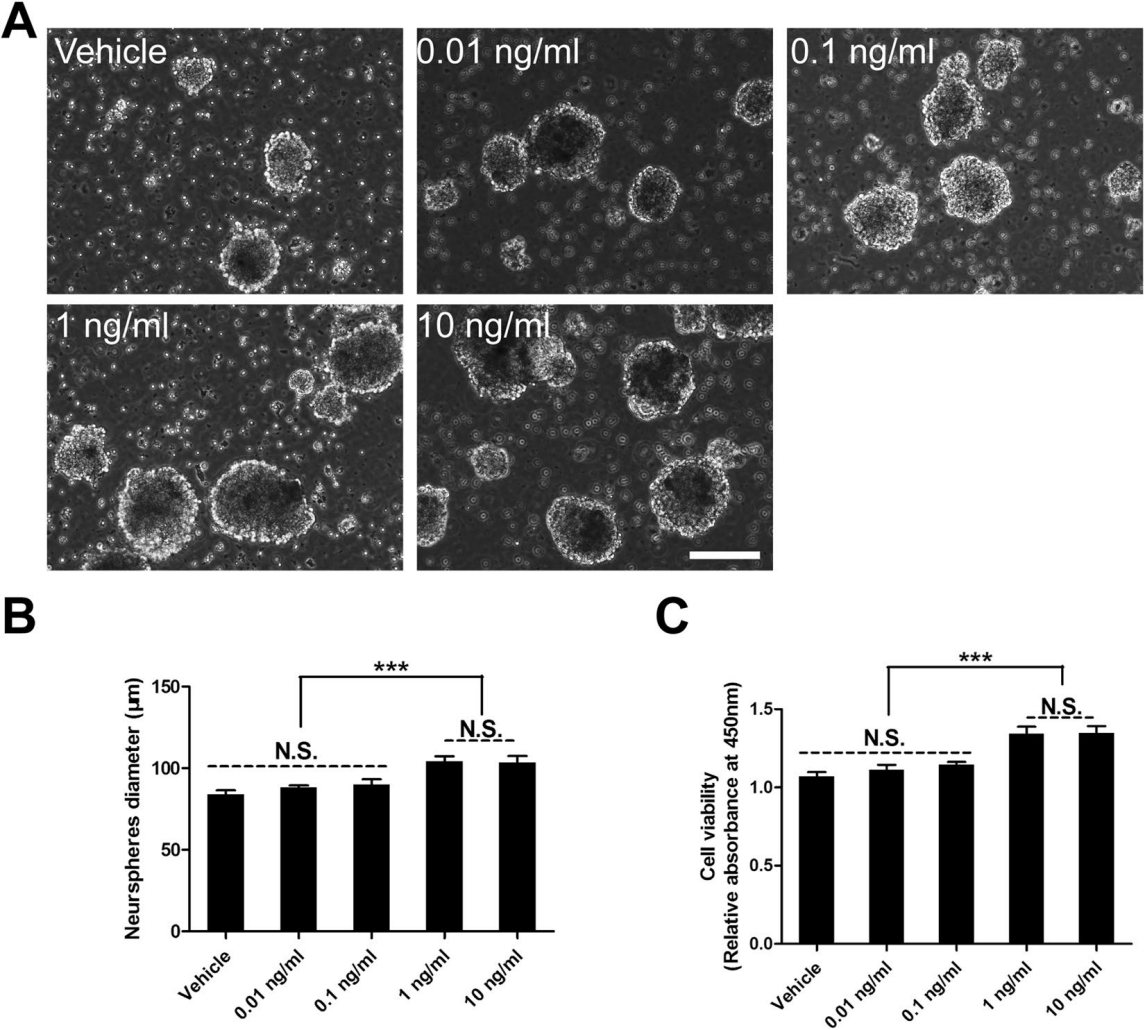
The suitable concentration of HMGB1 on NSCs proliferation. **(A)** The suspended growth of neurospheres incubated in various concentration of HMGB1 after 3 days. **(B)** Summarized graph from A showed the diameter of neurospheres in HMGB1. ***P < 0.01, one-way ANOVA followed by Tukey’s post hoc test (n = 4 for each group). **(C)** Bar graph showing the proliferation of NSCs growing in HMGB1 using CCK8 assays on day 3. ***P < 0.01, one-way ANOVA followed by Tukey’s post hoc test (n = 6 for each group). N.S. indicates no significant difference. Scale bar: 100 µm.

To investigate the effect of HMGB1 on NSCs, neurospheres were incubated in various concentration of HMGB1, and the diameter of neurospheres were determined using phase-contrast microscopy after 3 days. The results indicated the diameter of neurospheres were much larger in 1 ng/ml and 10 ng/ml groups than that in other groups (Fig. 1A,B). Next, the CCK8 assays were carried out to assess the effect of HMGB1 on NSCs proliferation. The data illustrated that the absorbance at 450 nm was significantly higher in 1 ng/ml and 10 ng/ml groups than that in other groups (Fig. 1C). Together, these data indicated that HMGB1 could promote NSCs proliferation in a dose-dependent manner. Hence, the dose of HMGB1 used was 1 ng/ml in the present study, which was in accordance with previous study^17^.

### HMGB1 evidently promoted NSCs migration via filopodia formation

**Figure 2:**
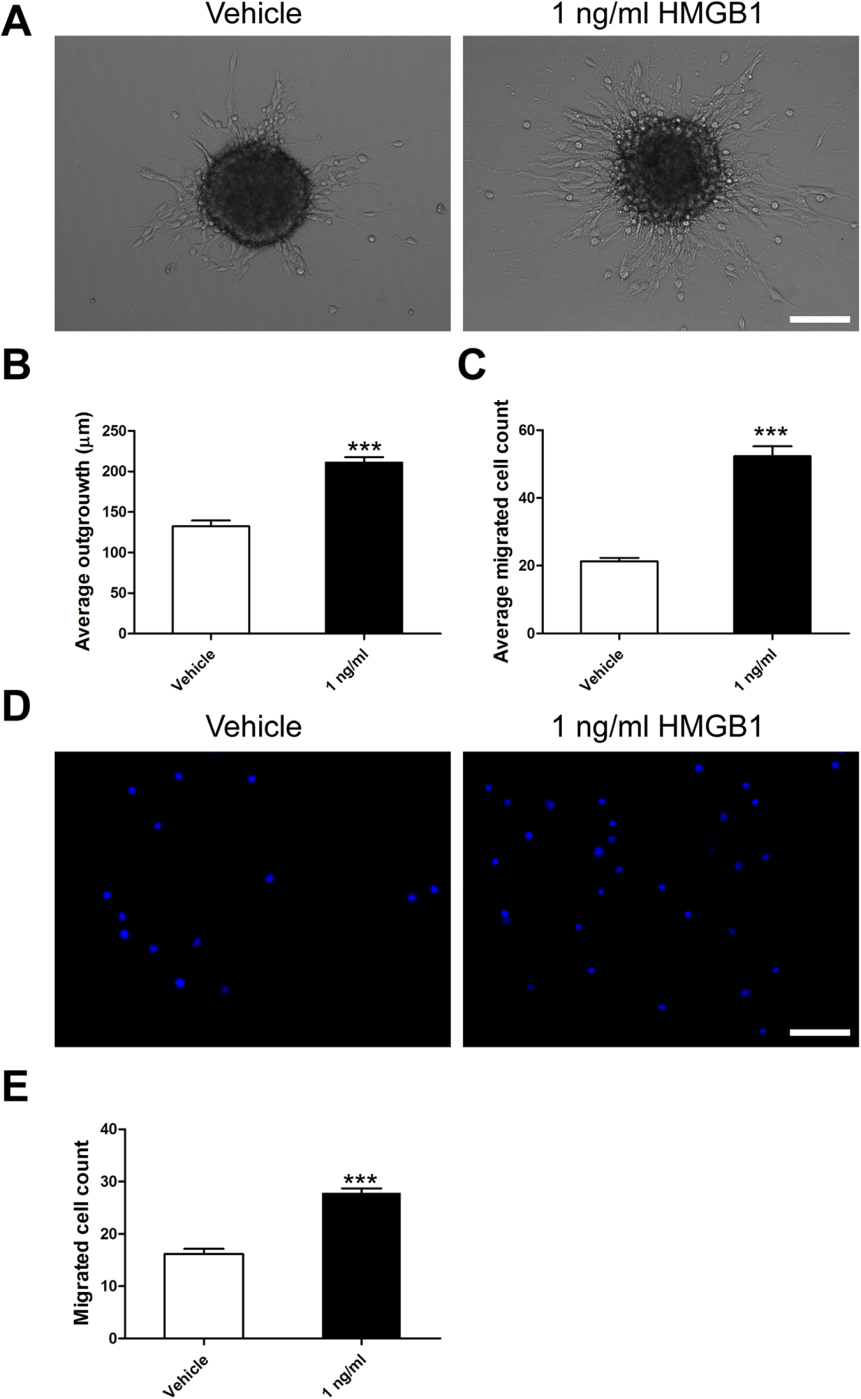
HMGB1 (1 ng/ml) promoted migration of NSCs. **(A)** Neurospheres were incubated in vehicle or 1 ng/ml HMGB1 with PLO pre-coated 24-well plates and images were captured by phase contrast microscopy after 24 hours. **(B)** Quantitative analysis of migration distance from neurospheres in vehicle or 1 ng/ml HMGB1, respectively. ***P < 0.01, Mann-Whitney U test (n = 4 for each group). **(C)** Bar graph showed the number of migration cells from neurospheres in vehicle or 1 ng/ml HMGB1, respectively. ***P < 0.01, Mann-Whitney U test (n = 4 for each group). **(D)** Transwell assays to evaluated the migration potential of NSPCs in vehicle or 1 ng/ml HMGB1. **(E)** Quantitative analysis indicated the number of migration cells from upper chambers to lower ones in vehicle or 1 ng/ml HMGB1, respectively. ***P < 0.01, Mann-Whitney U test (n = 4 for each group). Scale bar: 100 µm.

**Figure 3:**
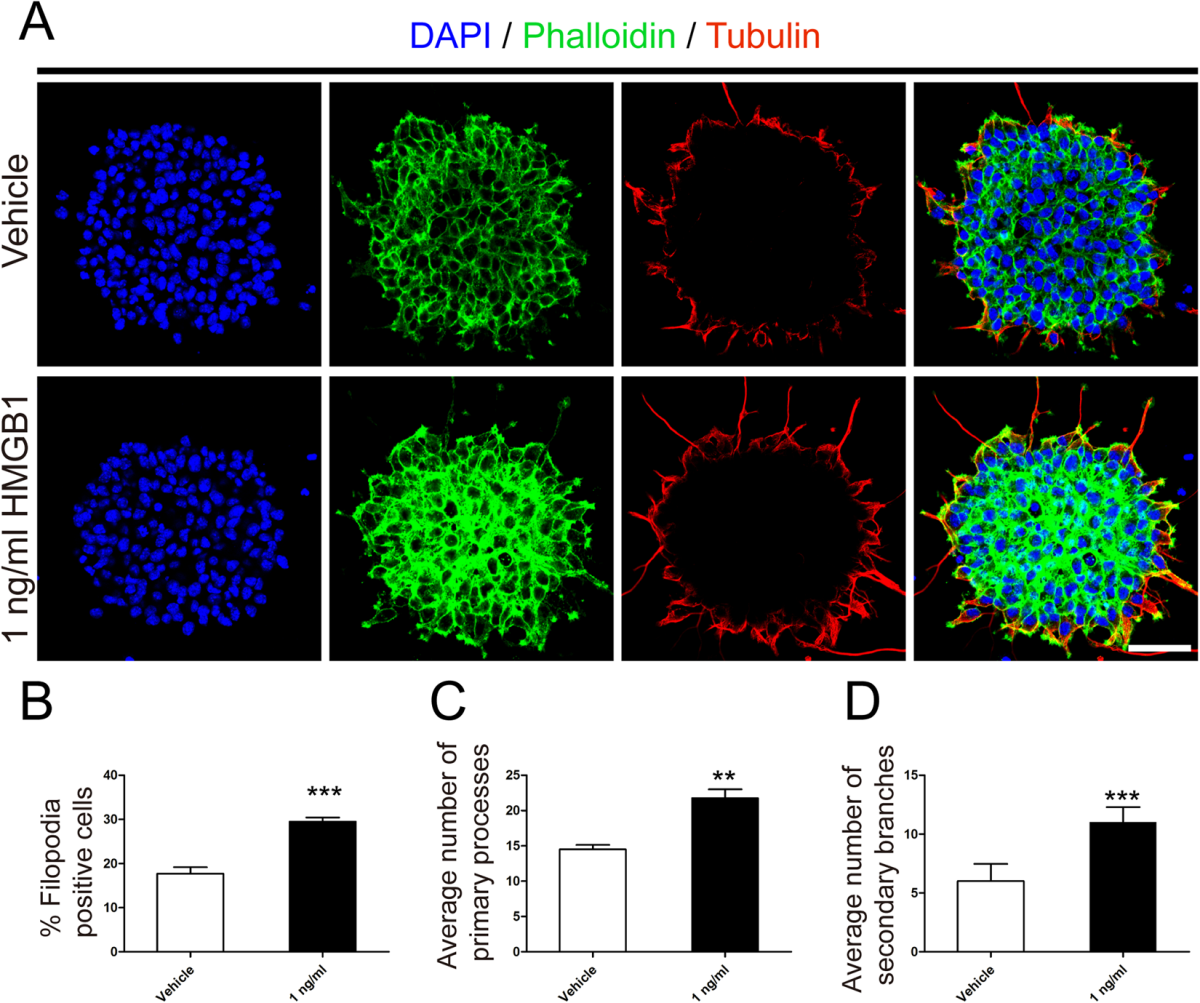
HMGB1 (1 ng/ml) facilitated filopodia formation. **(A)** Phalliodin staining (green) evaluated the nubmer of filopodia formation and tubulin immunostaining (red) indicated the cage-like microtubule structure in neurospheres in vehicle or 1 ng/ml HMGB1, respectively. Cell nuclei were stained with DAPI in blue. Scale bar: 20 µm. **(B)** Quantitative analysis showed the percent of filopodia formation in vehicle or 1 ng/ml HMGB1. ***P < 0.01, Mann-Whitney U test (n = 4 for each group). **(C)** Quantitative analysis of average number of primary leading processes in vehicle or 1 ng/ml HMGB1, respectively. ***P < 0.01, Mann-Whitney U test (n = 4 for each group). **(D)** Quantitative analysis of average number of secondary branches in vehicle or 1 ng/ml HMGB1, respectively. ***P < 0.01, Mann-Whitney U test (n = 4 for each group). Scale bar: 20 µm.

Given that 1 ng/ml HMGB1enhanced NSCs proliferation, we next checked the effect of HMGB1 on NSCs migration. NSCs were plated on PLO in the enrichment medium with or without 1 ng/ml HMGB1. First, the amount and distance of migrated NSCs were calculated under phase-contrast microscopy. Results showed the distance and number of NSCs in 1 ng/ml HMGB1 migrating from neurospheres were greatly increased than that in vehicle (Fig. 2A–C). Next, transwell assays were employed to ascertain the results gathered from above experiment. The data indicated that 1 ng/ml HMGB1 increased the mobility of NSCs (Fig. 2D,E). Together, these data suggested that HMGB1 hold the capacity to enhance NSCs migration. To understand why 1 ng/ml HMGB1 enhanced NSCs migration, we performed phalloidin staining to evaluate the filopodia formation, an indicator of cell polarization at the beginning of migration^19^. Meanwhile, we stained the expression of tubulin, a symbol of cage-like microtubule structure^20^, to evaluate the morphological structure changes in NSCs. The results sheded light on NSCs in 1 ng/ml HMGB1 developed more filopodia formation (Fig. 3A,B). Moreover, the average number of processes of NSCs in 1 ng/ml HMGB1 significantly increased than that in vehicle, including leading processes (Fig. 3A,C) and secondary branches (Fig. 3A,D). These data demonstrated that HMGB1 enhanced NSCs migration through increasing filopodia formation.

### HMGB1 upregulated RAGE expression

**Figure 4:**
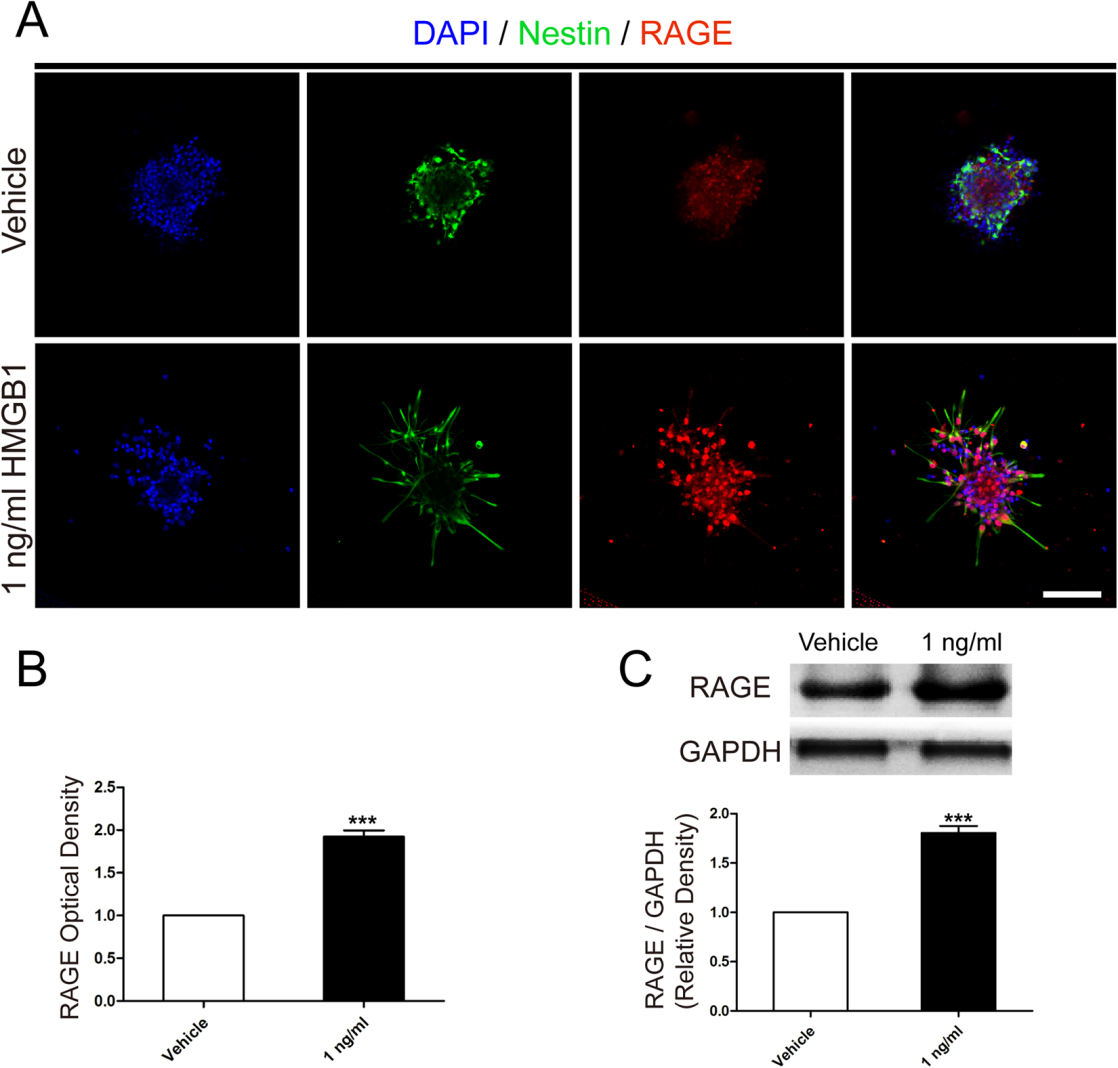
HMGB1 (1 ng/ml) upregulated RAGE expression. **(A)** The immunostaining images indicated RAGE expression (red) in vehicle or 1 ng/ml HMGB1. Cell nuclei were stained with DAPI in blue. Scale bar: 20 µm. **(B)** Bar graphs summarized semi-quantitative results from (**A**). ***P < 0.01, Mann-Whitney U test (n = 3 for each group). **(C)** Western blot assays indicated the different expression level of RAGE in vehicle or 1 ng/ml HMGB1 after migration for 24 hours. Bands were cropped from different parts of the same gel and analyzed using the Image Lab™ software for relative density and normalized to GAPDH control. ***P < 0.01, Mann-Whitney U test (n = 3 for each group). Scale bar: 100 µm.

RAGE is one of the main receptors for HMGB1 to guide many cell functions such as inflammation, apoptosis, or proliferation in tissue homeostasis and regeneration, especially in CNS^18^. Therefore, we hypothesized that HMGB1 activated RAGE to facilitate NSCs migration. To certify our hypothesis, we firstly evaluated different RAGE expression between vehicle and 1 ng/ml HMGB1 using immunofluorescence staining (Fig. 4A,B). The data illustrated NSCs in 1 ng/ml HMGB1 represented brighter optic density than that in vehicle. Next, western blotting assays were carried out to confirm the results obtained from immunofluorescence staining. The results indicated the same tendency as immunofluorescence staining (Fig. 4C).

### RAGE played a significant role in NSCs migration induced by HMGB1

**Figure 5:**
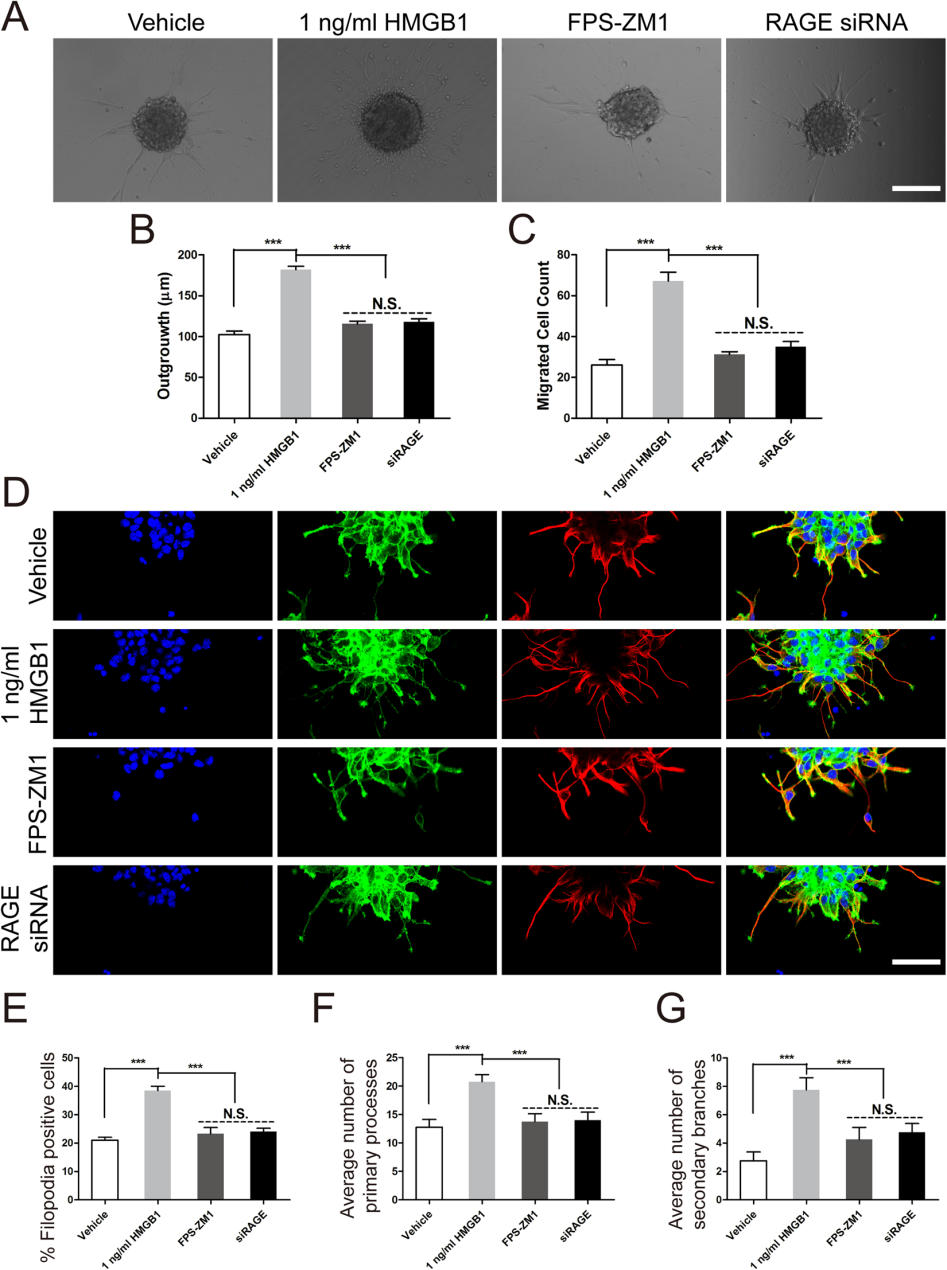
RAGE played a pivotal role in NSCs migration elicited by HMGB1. (**A**) The role of RAGE in NSCs migration induced by 1 ng/ml HMGB1. Neurospheres were incubated in vehicle, 1 ng/ml HMGB1, 50 nM FPS-ZM1, or siRAGE for 24 hours in PLO pre-coated 24-well plates and images were captured by phase contrast microscopy after 24 hours. Scale bar: 100 µm. (**B**) Quantitative analysis of migration distance from neurospheres. ***P < 0.01, one-way ANOVA followed by Tukey’s post hoc test (n = 3 for each group). (**C**) Summarized graph indicated the number of migration cells from neurospheres. ***P < 0.01, one-way ANOVA followed by Tukey’s post hoc test (n = 3 for each group). (**D**) The effect of RAGE on filopodia formation using phalliodin staining (green) and the cage-like microtubule structure with tubulin immunostaining (red) in neurospheres in different groups, respectively. (**E**) Bar graph indicated the percent of filopodia formation. ***P < 0.01, one-way ANOVA followed by Tukey’s post hoc test (n = 3 for each group). Scale bar: 20 µm. (**F**) Quantitative analysis of average number of primary leading processes. ***P < 0.01, one-way ANOVA followed by Tukey’s post hoc test (n = 3 for each group). (**G**) Quantitative analysis of average number of secondary branches. ***P < 0.01, one-way ANOVA followed by Tukey’s post hoc test (n = 3 for each group).

To further uncover the role of RAGE playing in HMGB1 resulting in NSCs migration, 50 nM FPS-ZM1, one of the RAGE antagonist, and RAGE-specific siRNA were used to detemine the role of RAGE playing in the NSCs migration indued by HMGB1. The data indicated that 50 nM FPS-ZM1 decreased the migrated distance and cell number after incubation for 24 hours (Fig. 5A–C). Furthmore, the downregulation of RAGE expression by siRNA were performed. The data indicated the same tendency as FPS-ZM1 (Fig. 5A–C). In addition, the phalloidin staining and tubulin immunostaining were performed to assess the role of RAGE in filopodia formation and morphological structure changes. The results indicated the percent of filopodia formation decreased greatly to the vehicle level with the treatment of FPS-ZM1 and RAGE-specific siRNA (Fig. 5D). Meanwhile, the bargraph demonstrated the decreased trend in filopodia formation (Fig. 5D,E), primary processes (Fig. 5D,F) and secondary branches (Fig. 5D,G). Taken together, these data indicated that RAGE played an evident role in facilitating NSCs migration induced by HMGB1 and the underlying mechnism was promoting filopodia formation.

## Discussion

In this study, the data ascertain the hypothesis that HMGB1 promotes NSCs migration via HMGB1/RAGE axis to promote filopodia formation. Meanwhile, results also indicate that 1 ng/ml HMGB1 enhances NSCs proliferation, which is in line with previous study^17^. NSCs, with the ability of multipotency and differentiation into three main subtypes of neural cells, hold promise for cell replacement therapy of many CNS injury, such as traumatic brain injury^21^, spinal cord injury (SCI) and stroke^22–24^. But the limited potency of proliferation, migration and differentiation into neuronal linage is one of the limitation restraining NSCs application after CNS injury.

HMGB1 facilitates NSCs activation. NSCs activation, including proliferation, migration and differentiation, helps promote neurogenesis in CNS disorders. Previous study has shown that HMGB1 could be passively released by necrotic or apoptotic cells and actively released from reactive astrocytes into the extracellular space to produce a higher concentration of local microenvironment after brain injury^11,25^. The nich with high HMGB1 concentration helps promote NSCs and endothelial cells proliferation to rebuild the injuried neurovascular network^11,12,14,17^. Furthermore, proper concentration of HMGB1 has been proved to enhance NSCs differentiation into neurons^18^. In this study, our results have indicated that 1 ng/ml HMGB1 promotes NSCs migration, which enlarge the experimental knowledge of HMGB1 on NSCs and the potential mechanism is HMGB1 binding with RAGE to enhance filopodia formation.

RAGE is a transmembrane protein, which is constitutively expressed during the embryonic stage, and decreased with time going on^18,26^. And, a variety of signalling molecules serve as ligands binding with RAGE to trigger signal transduction, such as advanced glycation end products (AGEs), HMGB1, S100/calgranulins, β-amyloid, phosphatidylserine, C3a and advanced oxidation protein products (AOPPs)^27^. Some researches have revealed that HMGB1 binding to RAGE triggers inflammatory cascade contributing to pathological effects after CNS injury^28,29^. While, the recent study demonstrates that HMGB1/RAGE axis plays a beneficial effect on NSCs migration. The reason why HMGB1/RAGE axis facilitates NSCs migration is partially due to filopodia formation. Filopodia, which are thin, finger-like and highly dynamic actin-rich membrane protrusions, can extend out from migrating cell edge and its extension could be mediated by numerous molecules, such as Rho family of small GTPases (including RhoA, Rac and Cdc42)^30^, ACTN4^19^, mDia^20^. Previous study has shown that Rac and Cdc42 could be activated to facilitate outgrowth of cultured cortical neurons after engagement of RAGE by a ligand^31^. Meanwhile, research also illuminnates that blockade of RAGE-amphoterin signalling interaction suppressing activation of p44/p42, p38 and SAP/JNK MAP kinases to inhibit tumour growth and metastases^32^. Hence, the underlying mechnism for HMGB1 promoting NSCs migration might be due to the activition of RAGE/Rac and Cdc42 or RAGE/MAPK signaling cascade.

There are also some other receptors for HMGB1like Toll-like receptor 2 (TLR2) and Toll-like receptor 4 (TLR4). TLR2, one of the membranous pattern recognition receptors, is one receptor for HMGB1. Study has indicated that HMGB1 exerts autocrine trophic effects on oligodendrocytes and myelin sheath under ischemic condition^33^. Meanwhile, TLR4 is another receptor of HMGB1. Research has shown that suppression of HMGB1/TLR4/NF-κB signaling pathway induces tissue repairment and motor function recovery attributing to inhibition of acute inflammatory injury post-SCI in rats^34^. However, previous study illuminates that activation of TLR 4 inhibits migration of mesenchymal stem cell (MSC) within 24 h^35^, which is not consistent with the present incubation of HMGB1 for at least 24 h. As a result, whether TLR2 takes part in the NSCs migration result from HMGB1 needs to be elucidated in our further work. However, the current study casts lights on RAGE plays a cardinal role in HMGB1 facilitating NSCs migration.

There are still a lot of work need to be done in the future to clarify the function of HMGB1 in promoting NSCs activation. First, the concentration of HMGB1, administrated by local injection or through intraperitoneal or intravenous injection, should be determined after brain or SCI injury. Second, the time window and duration of HMGB1 administration need to be further elucidated. In additon, our previous study has indicated that transplantation of NSCs with hypoxic preconditioning enhances expression and secretion of neurotrophic and growth factors (NT-3, GDNF and BDNF) to promote functional recovery after SCI in rats^36^. Hence, it might be a feasible approach to transplant NSCs prior to HMGB1 preconditioning to optimise cell replacement therapy.

In short, our present study has revealed pivotal effect of HMGB1 on NSCs migration and its possible underlying mechanism *in vitro*, which is a significant supplement of HMGB1 on NSCs activation. Together, our results demonstrate that HMGB1 could facilitate NSCs migration, which might serve as a candidate for CNS injury treatment and/or a preconditioning method for NSCs implantation.

## Materials and Methods

### Animal

This study was performed in accordance with t the China’s animal welfare legislation for the protection of animals used for scientific purposes and was approved by the local authorities of The Third Military Medical University for the care and use of laboratory animals (reference number: SYXK 2012-0002). Every effort was made to minimize the amount of animals and alleviate their sufferings. E14.5 C57BL/6 J mice were sacrificed after anesthesia with 2% isoflurane/air mixture (1–2 L/min).

### Primary Neural Stem Cells Culture

Primary NSCs from the cortices of fetal E14.5 C57BL/6 J mice pups were dissected and isolated as previously described^36^. Briefly, the cortices were washed twice in DMEM with 10% fetal bovine serum (FBS, vol/vol, GIBCO, Grand Island, NY) after incubation in 0.25% trypsin-EDTA (GIBCO, Grand Island, NY) at 37 °C for 30 min. Then, the tissue samples were triturated using a fire-polished Pasteur pipette and passed through a 100-µm Nylon cell strainer (BD Falcon, San Jose, CA) to collect the dissociated cell suspensions after they were washed twice with Dulbecco’s Modified Eagle’s Medium (DMEM, GIBCO, Grand Island, NY). Cell suspensions were cultured in DMEM/F12 medium supplemented with B27 (GIBCO, Grand Island, NY), 20 ng/ml EGF (Peprotech, Rocky Hill, NJ), 20 ng/ml FGF-2 (Peprotech, Rocky Hill, NJ) and 1% penicillin-streptomycin (vol/vol, Beyotime, Beijing, China) at 37 °C under humidified 5% CO_2_ condition as recommended. For passaging cells, neurospheres were harvested by centrifugation, dissociated in StemPro Accutase Cell Dissociation Reagent (GIBCO, Grand Island, NY) and grown in the medium described above. 50 nM FPS-ZM1 (Merck, White House Station, NJ) was used to block the physiological effect of RAGE^37^.

### Cell Proliferation Assay

First, the suspended growth of neurospheres were incubated in various concentration of HMGB1, and the diameter of neurospheres were determined using phase-contrast microscopy after 3 days. Next, Cell viability was assessed using a Cell Counting Kit-8 (CCK8, Dojindo, Tokyo, Japan), which determines the cell viability through WST reduction assay by detecting the dehydrogenase activity of viable cells. The cell suspension (100 μl, ~10000 cells/well) was dispensed in 96-well cell culture clusters with various concentration of HMGB1, and the cultures were incubated with 10% WST solution for 2.5 h at 37 °C. Then, the absorbance of the culture medium was determined using a microplate reader and a test wavelength of 450 nm, as well as a reference wavelength of 630 nm.

### Immunofluorescence Staining

For fluorescence staining, neurospheres, adhered to poly-L-ornithine (PLO) pre-coated coverslips, were fixed using 4% paraformaldehyde in 0.01 M phosphate-buffered saline (PBS, pH 7.4) for 30 minutes at room temperature and blocked with 5% v/v fetal bovine serum or with 0.3% v/v Triton-X 100 (Sigma-Aldrich, St. Louis, MO) in PBS. Neurospheres were incubated in mouse monoclonal to RAGE (1:200, Abcam, Cambridge, UK), goat polyclonal to Nestin (1:100, Santa Cruz Biotechnology, CA, USA), or mouse monoclonal to Tubulin (1:100, Beyotime, Beijing, China) overnight at 4 °C and then relative fluorescence secondary antibodies were incubated for 2 hours at room temperature. Cell nuclei were stained with 4′−6-Diamidino-2-phenylindole (DAPI, Beyotime, Beijing, China) for 10 minutes at room temperature. Samples were mounted onto glass slides, and images were captured using a confocal microscope (Carl Zeiss, LSM780, Weimar, Germany) and examined by Zen 2011 software (Carl Zeiss, Weimar, Germany).

### NSCs Migration Assay

For neurospheres migration assay, neurospheres were cultured in vehicle or 1 ng/ml HMGB1 with PLO pre-coated 24-well plates and the quantification methods were previously described^38^. Images were collected by phase contrast microscopy at 10× once every 2 h for 1 day allowing for the tracking of NSCs migration from neurospheres. Phase-contrast images (*n* = 4 per sample well) were analyzed using a custom-designed MatLab program (MathWorks, Inc., Natick, MA) as described previously and were normalized to baseline measurements taken 2 h after plating.

For single cell migration assays, NSCs were allowed to migrate through 8 μm pore Millicell cell culture inserts (Millipore, Temecula, CA) according to the manufacturer’s instructions as previously^39^. The upper chambers were seeded with 100 µl (1 × 10^5^) NSCs in vehicle or 1 ng/ml HMGB1. The lower chambers were filled with 600 µl DMEM/F-12 medium supplemented with 10% FBS which served as a chemoattractant. The NSCs were allowed to migrate from the upper to lower chambers for 24 hours at 37 °C in a humidified incubator with 5% CO_2_. Non-migratory cells were removed from the top of the membrane with a cotton swab and the cells attached to the lower surface of membrane were fixed in 4% paraformaldehyde at room temperature for 30 min and counterstained with DAPI, and the number was counted under fluorescence microscope. A total of 5 fields were counted for each filter.

### Western Blotting

Neurospheres in vehicle or 1 ng/ml HMGB1 were homogenized with RIPA (Sigma-Aldrich, St. Louis, MO) supplemented with protease inhibitor cocktail (Roche, Indianapolia, IN, USA). The protein concentration was measured using BCA Protein Assay Kit (Beyotime, Beijing, China). Proteins (20 µg/lane) were separated by 10% SDS-PAGE and electroblotted to polyvinylidene difluoride membranes (Roche, Indianapolia, IN, USA). After membranes were blocked in 5% skimmed milk in TBST at room temperature for 2 hours. Then, they were incubated with mouse monoclonal to RAGE (1:1000, Abcam, Cambridge, UK) and mouse monoclonal to GAPDH (1:1000, Zsgb-bio, Bejing, China) overnight at 4 °C. After incubation with peroxidase-conjugated (HRP)-conjugated secondary IgG (Zsgb-bio, 1:5000) for 2 hours at room temprature. All membranes were detected by ChemiDoc™ XRS^+^ imaging system (Bio-Rad, California, USA) using the WesternBright ECL Kits (Advansta, Menlo Park, CA, USA). Densitometric measurement of each membrane was performed using Image Lab™ software (Bio-Rad, California, USA). GAPDH, an internal control, was used to normalize the expression level of each protein.

### Filopodia Detection

To evaluate filopodia formation, samples were incubated in 488-phalloidin reagents (Life Technologies, Waltham, MA, USA). Then, coverslips were mounted in the ProLong Gold Antifade Reagent with DAPI (Sigma-Aldrich, St. Louis, MO), and images were visualised with a confocal microscope (Carl Zeiss, LSM780, Weimar, German) and analyzed using Zen 2011 software (Carl Zeiss, Weimar, Germany).

### RAGE siRNA Transfection

RAGE-specific siRNA (sc-36375) was purchased from Santa Cruz Biotechnology (CA, USA). The transfection of RAGE siRNA was performed as previously described^19^. Shortly, Lipofectamine RNAiMAX (Lipo, Invitrogen, Waltham, MA, USA) transfection reagent was used according to the manufacturer’s instructions. Meanwhile, the same amout of Lipofectamine RNAiMAX was used as negative control.

### Statistical Methods

All data were represented as mean ± SEM and statistical analyses were performed using SPSS v19.0 (SPSS Inc, Chicago, IL). Statistical significance was defined using One-way ANOVA followed by Tukey’s post hoc test or Mann-Whitney U test. A p < 0.05 was considered statistical significance.
